# ASAS-NANP Symposium: Mathematical modeling in animal nutrition: revolutionizing animal farming with artificial intelligence: trends, challenges, and opportunities

**DOI:** 10.1093/jas/skaf441

**Published:** 2025-12-18

**Authors:** Isabella C F S Condotta, Luis O Tedeschi

**Affiliations:** Department of Animal Sciences, University of Illinois, Urbana, IL, 61801; Department of Animal Science, Texas A&M University, College Station, TX, 77843

**Keywords:** digital agriculture, precision livestock farming, sensor integration, smart farming

## Abstract

Artificial intelligence (AI) can transform livestock farming as producers start using data-driven decisions in key areas, such as animal health, reproduction, behavior, nutrition, and production management. This review examines how AI technologies, like machine learning, computer vision, and sensor-based systems, help monitor and manage livestock more precisely, efficiently, and responsively. From early disease detection and estrus prediction to real-time behavior tracking and automated feeding systems, AI offers powerful tools for improving productivity, enhancing animal welfare, and supporting sustainable farm operations. Despite the promising technological advances, adopting AI in livestock systems comes with significant challenges. These include issues related to data quality and availability, model generalizability, infrastructure limitations, and ethical concerns involving data privacy and animal welfare. This review critically examines these obstacles and points out the need for robust, interpretable AI solutions that can adapt to specific farm conditions and offer meaningful explanations to end-users. Emerging trends like multimodal sensor fusion, digital twins, edge AI, and the integration of AI with genomics and climate data offer exciting possibilities for next-generation livestock management and smart farming systems. It is equally crucial to focus on human-centered design, participatory design, and group model-building approaches to ensure AI tools are accessible, trusted, and address the real needs of farmers and caregivers. This article explores AI’s potential to change livestock farming while advocating for interdisciplinary collaboration, inclusive innovation, and responsible deployment. It synthesizes current applications, challenges, and research frontiers. Ultimately, AI’s impact on animal agriculture depends on technical advancements as well as our ability to integrate these tools into systems that are biologically sound, socially accepted, and ethically responsible.

## Teaser

This review examines how artificial intelligence is reshaping livestock management through applications in health monitoring, reproduction, behavior analysis, and precision feeding. It highlights the current capabilities of AI systems, examines technical and ethical challenges, and outlines emerging research opportunities that can advance both animal science and data-driven agriculture.

## Introduction

The global livestock industry is transforming amid increasing demands for productivity, animal welfare, environmental sustainability, and labor efficiency (Niloofar et al., 2021). Traditionally, monitoring of animal health, reproduction, and nutrition depended on human observation, manual records, and periodic interventions. However, increasing system complexity, larger operation scales, and societal expectations for transparency and animal well-being now require more precise, data-driven approaches (Thumba et al., 2020). This shift marks the emergence of precision livestock farming (PLF), which integrates real-time data and automated technologies to enhance animal management (Berckmans, 2017).

Among the enabling technologies in PLF, artificial intelligence (AI) stands out as a transformative tool. AI encompasses machine learning (ML), computer vision (CV), and other computational techniques that enable machines to analyze data, recognize patterns, and make informed decisions (Fuentes et al., 2022; Melak et al., 2024). In livestock systems, AI technologies are increasingly employed to identify early signs of disease from video or sound data, detect estrus from behavioral cues, estimate body weight from images, and adjust feeding strategies based on real-time intake patterns (García et al., 2020). These applications rely on the convergence of enabling technologies, including the Internet of Things (IoT), wearable and non-invasive sensors, thermal and multispectral imaging, cloud computing, and real-time analytics platforms.

The potential of AI in livestock systems is substantial. For instance, ML algorithms can now process thousands of data points per animal daily, providing unprecedented insights into individual and herd-level behavior (McVey et al., 2023). Many CV systems have demonstrated the capability for early disease detection, enabling proactive management and supporting earlier interventions (Okinda et al., 2019; Jorquera-Chavez et al., 2021; Parikh et al., 2024). Similarly, audio analysis technologies effectively differentiate coughing patterns or vocalizations associated with stress or respiratory illness (Cordeiro et al., 2013; Carpentier et al., 2018; Wang et al., 2024).

Despite these promising developments, the implementation of AI in livestock systems continues to face significant challenges. The diversity of livestock environments, ranging from large commercial operations to smallholder farms, makes it difficult to standardize data collection and deploy robust AI systems. Additionally, ethical and legal concerns regarding data privacy, algorithmic bias, and displacement of traditional labor roles require careful consideration. Furthermore, technical challenges such as sensor reliability, data quality, and model generalizability continue to hinder the widespread adoption of these technologies (Georgopoulos et al., 2023; Kaushik et al., 2024).

This literature review summarizes current knowledge on the integration of AI in livestock farming systems. It examines core AI applications in the domains of health, reproduction, behavior, nutrition, and production, highlighting emerging trends in multimodal sensing, edge computing, and digital twin technologies. It also discusses persistent challenges, including limited data availability, model interpretability, infrastructure constraints, and stakeholder adoption. It then outlines future research opportunities and proposes pathways toward scalable, responsible, and inclusive implementation of AI in livestock farming.

The objectives of this article are to provide background on AI technologies and their relevance to livestock farming, including a historical perspective; to explore current AI applications across key livestock management domains, with emphasis on real-world implementations and recent scientific developments; to examine significant challenges and barriers to adoption, spanning technical and operational constraints as well as ethical and social implications; and to discuss emerging trends and innovative research directions, followed by a conclusion and future outlook.

## Background and Technological Foundations

### Overview of artificial intelligence in agriculture

Artificial intelligence refers to computational systems capable of performing tasks that typically require human intelligence, including learning from data, recognizing patterns, making predictions, and solving problems. Machine learning, a subset of AI, enables algorithms to learn from data, identify patterns, and adapt their outputs without explicit rule-based programming. This allows systems to improve performance with experience. Deep learning (DL), an advanced subset of ML, employs artificial neural networks (ANNs) to model complex, hierarchical patterns, making it well suited to image and sound recognition tasks common in agricultural monitoring (Kamilaris and Prenafeta-Boldú, 2018). Computer vision is another essential subfield of AI that enables automated interpretation of visual data, such as images or videos, to monitor livestock behavior, identify individuals, or detect signs of illness (Liu et al., 2020; McDonagh et al., 2021; Han et al., 2023; Islam et al., 2023).

In agricultural systems, AI processes large and heterogeneous data streams obtained from sensors, cameras, microphones, and other digital devices (Tedeschi et al., 2021). A key strength of AI is its ability to detect complex, often nonlinear relationships in large, multidimensional datasets that are invisible to human observers or traditional statistical approaches. For example, AI systems can continuously monitor herds without human intervention and flag animals that deviate from normal patterns of activity, feeding, vocalizations, and posture, for example.

Many agricultural AI systems employ several learning paradigms. Supervised learning, where models are trained on labeled data, is commonly used for classification tasks such as identifying lameness or forecasting feed intake. In contrast, unsupervised learning explores unlabeled data to detect latent behavioral patterns, group animals with similar activity profiles, or flag anomalies. Although still emerging in livestock applications, reinforcement learning enables adaptive systems, such as autonomous feeders, to learn optimal strategies as they interact continuously and receive feedback.

The efficacy of AI systems in agriculture depends on a supporting technology ecosystem. The IoT could integrate wearable sensors, automated feeders, environmental monitors, and cameras, enabling continuous, real-time data collection and monitoring. Edge computing could enhance data processing directly on the farm or on devices, reducing latency and enabling prompt interventions. For example, low-power devices installed in poultry houses or barns could process temperature, sound, and activity data locally, triggering immediate alerts without relying on cloud connectivity. Cloud computing can complement edge solutions with scalable storage and robust analytics, enabling integration and analysis of data from multiple sources or farms. Moreover, 5G and other wireless connectivity advancements, such as LoRaWAN, could further enhance real-time data transmission, which is essential for remote or extensive farming operations.

Together, these technologies form the infrastructure for successful AI implementation in livestock farming systems. However, reaching their full potential requires careful integration that ensures interoperability and alignment with animals’ biological and behavioral complexities, and with real-world farming challenges.

### Evolution of AI in livestock systems

The adoption of AI in livestock farming has evolved from manual observation tools to increasingly automated and intelligent systems. This trajectory helps contextualize current and emerging applications. Initial implementations of PLF technologies primarily relied on radio-frequency identification (RFID) tags, automated weighing systems, and basic alert systems that flagged abnormalities such as ventilation failures or reduced water intake (Berckmans, 2006).

As technological capabilities advanced, real-time sensor-based systems became more common. Devices such as accelerometers, thermal cameras, global positioning system (GPS) trackers, and microphones enabled continuous, individual-level monitoring of livestock behavior and physiology. For example, accelerometers have been used to monitor feeding and locomotion in dairy cows (Vázquez Diosdado et al., 2015; Beer et al., 2016; Barker et al., 2018; Werner et al., 2019; Balasso et al., 2021; Iqbal et al., 2021), while thermal imaging has enabled early detection of disease and mastitis (Schaefer et al., 2012; Zhang et al., 2020; Anagnostopoulos et al., 2021; Wang et al., 2022a; Gayathri et al., 2024).

By the 2010s, ML and CV began to gain traction in animal agriculture. ML algorithms demonstrated value in tasks such as predicting tail-biting outbreaks in pigs (Domun et al., 2019; Larsen et al., 2019; Ollagnier et al., 2023) and monitoring rumination patterns of cows (Hamilton et al., 2019; Ayadi et al., 2020; Abdanan Mehdizadeh et al., 2023; Li et al., 2024a). Convolutional neural networks (CNNs), a class of DL models, were applied successfully to behavior recognition tasks, including detecting lying, feeding, and mounting in cattle and pigs (Li et al., 2019; Achour et al., 2020; Alameer et al., 2020; Fuentes et al., 2020; Chen et al., 2020a; Yu et al., 2022). Additionally, CV models have shown high accuracy for estimating livestock body weight, providing a non-invasive alternative to traditional weighing systems that rely on scales (Ma et al., 2024a).

The rise of multimodal sensing systems has further expanded AI capabilities. Researchers have reported stronger robustness and accuracy when data from multiple sources are combined, such as audio, thermal, and 3D video inputs. For example, studies have used multimodal data, including audio and images, to improve the detection of respiratory diseases in pigs (Ji et al., 2022; Chae et al., 2024). In dairy systems, multi-sensor approaches have enabled detection of metabolic disorders, oestrus, and behavior (Holman et al., 2011; Sturm et al., 2020; Tian et al., 2021; Arablouei et al., 2023).

Despite the growing body of evidence supporting the efficacy of AI in livestock systems, adoption remains variable across farm sizes and regions. Larger operations often possess the infrastructure and capital necessary to implement and maintain advanced technologies. At the same time, smaller farms and ranches may face barriers such as high costs, a lack of digital literacy, and limited access to data interpretation tools. Moreover, variability in environmental conditions, animal genetics, and housing systems across production sites limits the generalizability of AI models and requires site-specific calibration and validation.

Nonetheless, AI research in animal agriculture is expanding rapidly, with open-access datasets, advances in sensor design, and interdisciplinary collaborations accelerating progress. For instance, research increasingly focuses on making models more interpretable and accessible to producers through user-friendly interfaces and the incorporation of domain expertise into algorithm development (Sykes et al., 2022; Mallinger et al., 2024; Neethirajan et al., 2021), including the development of hybrid intelligent mechanistic models (HIMM). These models combine AI’s pattern recognition capabilities with biologically based mechanistic models to enhance explainability and robustness (Tedeschi, 2019, 2022, 2023).

## Current Applications of AI in Animal Farming

Artificial Intelligence has emerged as a transformative tool in livestock production systems. It enables real-time, non-invasive monitoring and supports data-driven decision-making. Validated AI applications now cover animal health, reproduction, behavior, feeding, identification, and integrated farm management. These systems increasingly rely on ML and DL to process complex datasets from video, audio, thermal imaging, and wearable or environmental sensors. Table 1 provides a structured overview of AI applications in key areas of animal farming.

**Table 1.** Overview of AI applications across animal farming domains

Domain	Input modality	Key applications
**Health Monitoring**	Milk yield, SCC, conductivity, activity, thermal images, audio	Mastitis detection, lameness detection, pain/facial analysis, fever detection, respiratory illness detection
	Video footage, posture, accelerometry	Gait and body condition monitoring
**Reproduction & Estrus Detection**	Video (mounting, locomotion), thermal IR, pose estimation	Estrus prediction, reproductive cycle monitoring
	Audio recordings (vocalization)	Estrus-associated vocalization classification
	Multimodal fusion (video + thermal + audio)	Enhanced estrus and farrowing detection
**Behavior & Welfare Assessment**	Video, audio, accelerometers, facial images, positioning sensors	Aggression detection, social network analysis, grimace scales, emotional state monitoring
	Multimodal systems (CV + audio + motion)	Welfare tracking and stress monitoring
**Nutrition & Precision Feeding**	RGB-D cameras, audio (pecking/chewing), GPS, LPS, accelerometers	Feed intake estimation, feeding behavior classification
	CV and audio combined with growth tracking	Growth-based feed adjustment systems
**Productivity Monitoring**	RGB, 3D, or depth cameras, body dimension extraction, milk/egg data	Weight prediction, milk yield anomaly detection, egg grading
	Video-based monitoring systems	Egg counting, defect detection
	Sensor + ML integration	Production forecasting, anomaly detection

### Animal health monitoring

Animal health is foundational to sustainable and profitable livestock production, and the early identification of disease is crucial for minimizing treatment costs, preventing outbreaks, and improving animal welfare. Traditional methods, such as visual inspection or threshold alarms from isolated sensors, often detect conditions too late for optimal intervention. AI approaches provide a transformative upgrade to these systems. They integrate multimodal sensor data and automatically detect patterns or anomalies associated with health deterioration. enabling continuous, remote, and scalable health monitoring across species and housing systems.

A key area of research has been the detection of mastitis, a prevalent and costly disease in dairy cattle. Studies have demonstrated that ML algorithms that integrate sensor data such as milk yield, somatic cell count, electrical conductivity, and behavior metrics like rumination time outperform traditional threshold methods. For example, Tian et al. (2024) reported that combining milk production and conductivity data using supervised ML models improved early detection of clinical mastitis. Similarly, Cavero et al. (2008) used an ANN to classify mastitis presence with promising results. A broader review by Ozella et al. (2023) noted that AI-based mastitis models are increasingly incorporated into automatic milking systems for real-time detection.

Lameness detection is another well-established application of AI. This condition is difficult to identify with visual observation in large or group-housed herds. Early work explored image-processing methods (Song et al., 2008; Condotta et al., 2020), and later studies integrated CV-based models to accelerate analysis and enable real-time use. Wu et al. (2020) applied a YOLOv3-based DL model to analyze top-view video data and identify dairy cows with abnormal gait patterns in real time. In pigs, Zhenbang et al. (2024) used a 3D CNN to classify gait sequences from video footage, achieving strong agreement with expert scoring. These systems enable consistent and objective evaluation of locomotor issues, making them well-suited for integration into automated management platforms.

Beyond locomotion, AI has also been applied to evaluate health-related physical indicators, such as body condition and pain expression. Çevik (2020) demonstrated the use of DL to automatically classify body condition scores from images of dairy cows, offering a non-invasive and repeatable alternative to manual scoring. Additionally, facial recognition models using CNNs have been trained to detect pain in sheep based on ear posture, eye changes, and muscle tension (Noor et al., 2020). These approaches are promising for welfare monitoring but require broader validation across species and environments.

Audio-based disease monitoring has also been successfully implemented. Respiratory diseases often manifest through coughing or sneezing before more visible symptoms appear. Chae et al. (2024) developed a multimodal DL system using CNNs and recurrent neural networks (RNNs) to detect cough events in pigs accurately. Likewise, Schaefer et al. (2012) demonstrated that infrared thermography could detect early respiratory infections in calves and identified increased eye and nasal temperatures as early indicators. This finding supports integration of multimodal approaches, such as combining visual and acoustic signals, into AI-based systems.

This integration of multimodal data, including video, audio, thermal, and motion sensor streams, is in early stages of study to further enhance the robustness of AI-based health diagnostics. For example, Dhaliwal et al. (2025) demonstrated that combining video and audio improved early lameness detection in dairy cows, with fewer false positives than unimodal models. These fusion-based approaches can offer redundancy, which in AI systems means the duplication of critical components to increase reliability, safety, and fault tolerance under noisy or incomplete conditions.

Additionally, wearable sensor data, such as accelerometers, rumination monitors, or temperature tags, can be used in ML models to track early physiological deviations. These models have been used for a range of applications, including the prediction of metabolic disorders, fever detection, and monitoring of stress responses in cattle, swine, and sheep (Neethirajan, 2017; Jorquera-Chavez et al., 2021; Stygar et al., 2021).

While these technologies advance rapidly, current systems remain under development and are often limited to pilot or semi-commercial stages. Validation in large, diverse herds and different management systems remains essential for widespread adoption.

### Reproductive monitoring and estrus detection

Efficient and timely estrus detection is essential for maximizing reproductive success in animal farming. Accurate identification of the onset of estrus enables better insemination timing, improves conception rates, reduces hormone use, and minimizes labor associated with visual monitoring. Traditional methods, such as chalking, standing heat observation, or tail painting, are often subjective, labor intensive, and less effective in group-housed systems. Artificial intelligence, particularly systems powered by CV, acoustic analysis, and deep learning, can provide new tools for automated, continuous, and individualized estrus monitoring across species, with calibration often needed for different species and housing systems.

AI-driven CV technologies have been used to detect behavioral cues of estrus, including increased locomotion, standing reflex, and mounting behavior. For example, Li et al. (2019) developed a DL-based system that recognized mounting behavior in pigs using surveillance video footage. Küster et al. (2020) implemented CV to monitor changes in sow activity, showing that video-based behavior analysis can detect events related to estrus and farrowing. More recently, Lodkaew et al. (2023) introduced CowXNet, a DL framework for estrus detection in dairy cattle using visual behavior cues in group-housed systems, which effectively tracks individual cows within herd environments.

Thermal imaging has also been explored as a method for estrus prediction. Feng et al. (2019) demonstrated that infrared thermal cameras could detect temperature increases in sow vulvas, an indicator of estrus. They used partial least squares regression to predict rectal temperatures with a *R*^2^ of 0.80. If integrated with behavioral cues and CV systems, this approach could enhance the accuracy of estrus detection.

Multimodal AI systems that integrate data from various sensors, such as visual, motion, thermal, and audio, are increasingly being explored to enhance the robustness of livestock behavior monitoring under commercial conditions. For instance, Cai et al. (2025) developed a multimodal feature fusion method that combines audio and thermal infrared image data to improve the accuracy and robustness of estrus monitoring in breeding pigs. Additionally, Aryawan et al. (2024) proposed a novel approach using pose estimation with a deep learning model for real-time estrus detection in female cows. Furthermore, Arıkan et al. (2023) introduced a method that integrates estrus detection with cow identification for use with augmented reality (AR) devices, employing deep learning-based mounting detection and then the system identified the mounting region of interest (ROI) with a YOLOv5 model.

Acoustic signals associated with estrus, including specific vocalizations, have also been analyzed with AI. Jung et al. (2021) developed a CNN-based system to classify cattle vocalizations in real time using noise-filtered audio, achieving classification accuracy above 90%. While their system was not designed exclusively for estrus detection, similar acoustic features have been reported to correlate with estrus phases in pigs and cattle (Schön et al., 2007; Wang et al., 2022c, 2023) and could be combined with video or thermal inputs into multimodal monitoring tools.

Field-level validation of AI systems remains crucial for commercial adoption. Verhoeven et al. (2023) evaluated an AI-powered estrus detection system in sows using over 6,700 reproductive cycles across three farms. The system, which used overhead cameras and a behavior recognition algorithm, significantly improved farrowing rates and reduced repeat breedings at two of the farms under routine farm conditions.

Finally, fuzzy logic and ML models applied to sensor data have also performed well. Zarchi et al. (2009) developed a fuzzy logic-based model for estrus detection in dairy cows, achieving 85.3% sensitivity and 100% specificity using data on milk conductivity, activity, and yield. In a motion-based application, Aloo et al. (2024) trained an artificial neural network on accelerometer and temperature data to detect estrus in cattle, yielding an accuracy of 89.5%.

### Behavior and welfare assessment

Animal behavior serves as a crucial indicator of welfare status. Changes in postural activity, feeding frequency, rest patterns, and social interactions often precede overt signs of illness, pain, or stress. Traditional behavioral assessments rely heavily on human observation, which is subjective, intermittent, and impractical for large-scale or continuous monitoring. Artificial intelligence enables automated, scalable, and real-time behavioral assessments in livestock production systems when combined with sensor technologies such as video, wearables, and microphones.

Computer vision and DL models have been widely used to monitor behaviors such as lying, standing, walking, and feeding. Nasirahmadi et al. (2019) developed a system using image processing and machine learning to automatically classify pig postures from overhead images, enabling real-time tracking of activity in group-housed environments. Cowton et al. (2019) designed a DL pipeline capable of identifying and tracking individual pigs, extracting behavior metrics like location, movement, and feeding duration.

To capture temporal patterns in behavior, CNNs have been combined with long short-term memory (LSTM) architectures. Chen et al. (2020b) employed a CNN–LSTM model to analyze video footage of pigs, aiming to identify aggression episodes. Their system achieved high classification accuracy (97.2%), demonstrating how the combination of spatial and temporal features could enhance behavior detection under commercial housing conditions.

Advanced CV models, such as instance segmentation, enable the identification of multiple animals in the same frame, even under occlusion. Hu et al. (2021), for example, proposed a dual attention-guided feature pyramid network for segmenting and tracking pigs in dense pen environments. These methods are particularly useful in swine and poultry systems where animals often overlap in camera views.

AI approaches have also been developed to monitor social behaviors and group-level dynamics. Social network analysis (SNA) can be used to quantify affiliative and aggressive behaviors in livestock through analysis of proximity, co-occurrence, and interaction patterns derived from automated monitoring systems. Agha et al. (2025) demonstrated this approach with positioning data from pigs, revealing latent social structures within pens and offering insights into social hierarchy formation and individual variability in sociality.

Facial recognition and expression analysis have gained traction as tools for assessing pain and emotional states in farm animals, with the goal of supporting non-invasive, real-time welfare assessment across species. These methods rely on identifying specific facial action units, such as orbital tightening, ear position, and changes in the nose or mouth, that correlate with discomfort. Noor et al. (2020) trained convolutional neural networks to detect such features in sheep, resulting in a reliable and automated sheep grimace scale. In horses, Dalla Costa et al. (2014) developed the Horse Grimace Scale (HGS) to assess pain following routine castration, focusing on facial expressions like stiffly backward ears, orbital tightening, and tension around the eye area. Similarly, Di Giminiani et al. (2016) introduced the Piglet Grimace Scale to evaluate pain in piglets undergoing tail docking and castration, identifying specific action units, such as bulging cheeks and orbital tightening.

In addition to pain recognition, facial analysis has also been explored to assess emotional states. The WUR Wolf platform, developed by Neethirajan (2021), applies deep learning algorithms such as YOLOv3, YOLOv4, and Faster R-CNN to monitor facial features, including ear posture and eye white visibility, in cattle and pigs. When linked with other behavioral and physiological data streams, the platform targets broader welfare monitoring goals. The system achieved a classification accuracy of around 85% and was designed for real-time monitoring.

Wearable sensors, such as accelerometers, are widely used to monitor movement and activity in dairy cattle, pigs, and small ruminants. These devices can detect deviations from normal movement or lying behavior, which may indicate discomfort or illness. When paired with ML models, they enable automated behavior classification and facilitate longitudinal welfare monitoring. Fuentes et al. (2022) reviewed such systems, noting their scalability and high predictive performance in real-world applications.

Acoustic monitoring offers another promising avenue for assessing welfare. Animals vocalize differently in response to stress or pain, and AI models can accurately classify these vocalizations. Jung et al. (2021) developed a real-time vocal classification system for cattle using CNNs and noise-filtering preprocessing. Their system achieved classification accuracy of over 90%, demonstrating the potential for sound-based welfare indicators.

A review by Debauche et al. (2021) highlights that many AI techniques developed for behavior monitoring in one species can be generalized to others, particularly for common behaviors like grazing, lying, and locomotion. They emphasize the benefits of combining multiple sensors, such as accelerometers, video, and microphones, to improve classification accuracy. The placement of sensors and the selection of appropriate data processing algorithms are also critical for system performance. Additionally, trends such as edge computing are enabling real-time behavior analysis directly on the farm, reducing data transmission costs and latency.

The integration of multimodal systems is becoming increasingly common. These systems improve detection robustness under varying environmental conditions and animal behaviors. Wang et al. (2022c) and Fuentes et al. (2022) emphasize that future systems are likely to rely on DL architectures capable of processing multimodal inputs for enhanced welfare analysis.

### Precision feeding and nutrition

Feeding represents the most significant variable cost in livestock production, making feed efficiency and precision nutrition vital for economic and environmental sustainability. AI technologies have emerged as powerful tools to individualize feeding strategies based on real-time and historical data on intake behavior, growth, physiological status, and activity patterns. These approaches reduce feed waste, improve animal performance, and help minimize environmental impacts such as methane emissions from enteric fermentation.

AI-powered systems are used to estimate feed intake, support individualized feeding optimization, and predict feeding behavior using various sensor modalities. In dairy cattle, Bezen et al. (2020) developed a CV system utilizing RGB-D cameras and DL algorithms to estimate individual cow feed intake with high accuracy. Additionally, Bloch et al. (2021) proposed a system to measure individual cow feed intake in commercial dairies that used CV for individual cow identification. These studies exemplify AI’s ability to support site-specific feeding decisions, and they enable dynamic diet formulation for enhanced efficiency. Additionally, predictive models could incorporate factors such as milk production, body weight, lactation stage, and environmental conditions to estimate daily nutrient requirements and inform ration adjustments, which supports more responsive feeding management.

Multimodal systems that combine video, audio, and accelerometer data have also shown promising results. Barker et al. (2018) employed a combination of local positioning systems (LPS) and accelerometers to quantify feeding behavior in lame versus non-lame dairy cattle, which enables the early identification of animals deviating from normal feeding patterns. In extensive grazing systems, wearable GPS collars and accelerometers have been deployed to track livestock location and activity. Machine learning algorithms, particularly Random Forest classifiers, have been used to distinguish between grazing, walking, resting, and ruminating behaviors. For example, Williams et al. (2016) employed GPS data and ML techniques to model pasture use in dairy cows, showing high predictive accuracy for spatial behavior analysis.

Poultry operations are starting to benefit from AI applications that monitor feed intake and assess growth. Vision systems using depth cameras and CNNs have been developed to recognize feeding behavior and estimate body size in crowded environments. For instance, Guo et al. (2022) demonstrated that video-based models can detect broiler feeding behavior with high precision, highlighting the potential of non-invasive tools for monitoring flock-level patterns. While daily tracking and individualized feed adjustments remain under development, these tools provide valuable insights that can support more responsive management strategies. In broiler systems, Aydin et al. (2015) introduced a sound-based monitoring tool capable of estimating feed intake using audio signals from pecking behaviors. The model distinguished feeding activity in real-time, offering a potentially scalable, non-invasive method to track consumption across multiple animals simultaneously.

In swine production, real-time growth monitoring using CV models has the potential to inform feeding interventions. Chen et al. (2020a) employed a video-based deep learning model to detect and quantify feeding time in pigs, distinguishing individual behaviors, such as feeding, drinking, and idling, from overhead video footage. Systems like those presented by Cang et al. (2019) estimate pig weight patterns without interrupting animal routines, and can enable adaptive feed delivery based on projected growth trajectories.

Overall, AI advances livestock feeding and enables data-driven decisions tailored to the biological needs of individual animals or groups, with potential benefits for productivity, animal welfare, and carbon-footprint reduction. AI-based precision feeding enhances feeding efficiency and reduces nitrogen oversupply, which decreases waste and limits excess nitrogen and phosphorus excretion, which are key contributors to ammonia and nitrous oxide emissions from manure management (Pomar et al., 2021). Improved nutrient use efficiency is also linked with environmental sustainability; for example, recent lifecycle assessments have shown that precision feeding strategies can lower global warming potential as they reduce feed inputs per unit of animal product (Llorens et al., 2024). Feed-crop production (including fertilizer, land-use change, and transport) and enteric methane emissions are among the largest contributors to greenhouse-gas emissions in ruminant livestock systems (Grossi et al., 2019). As a result, even modest gains in feed conversion efficiency can reduce emission intensity.

### Production monitoring and management

Monitoring livestock productivity is crucial to effective farm management, as it informs decisions related to nutrition, marketing, reproduction, and health. While manual assessments of growth, milk yield, or egg production remain common, they are labor-intensive and often lack precision or timeliness. AI technologies have the potential to offer scalable, non-invasive alternatives for continuous productivity monitoring. These tools support individualized management as they extract performance metrics from visual, acoustic, and environmental data streams.

One of the most widely studied AI applications in this domain is body weight estimation using computer vision. Accurate body weight is a critical productivity metric for beef, dairy, swine, and poultry systems; however, traditional weighing methods are time-consuming and stressful for animals. Multiple studies have demonstrated that CV-based approaches can automate weight estimation using RGB or depth images. For example, Condotta et al. (2018) predicted grow-finishing pigs’ weights from depth images using ANNs with MPE as low as 3.93% (*R*^2^ between predicted and actual weight of up to 0.99). Similarly, Cominotte et al. (2020) employed a Kinect depth camera combined with regression and neural networks to estimate body weight and average daily gain in beef cattle, achieving high predictive accuracy (*R*^2^ up to 0.92). Wang et al. (2021) reviewed digital image-based ML models across species and emphasized their utility in supporting management decisions such as optimal marketing time and detecting deviations from expected growth curves.

Condotta et al. (2020) emphasized the importance of considering the body weight, size, and conformation of modern animals when designing facilities and equipment, showcasing the use of depth imaging techniques to acquire dimensions of interest. Similarly, recent reviews have detailed advances in animal body dimension measurement techniques. Ma et al. (2024a) and Ma et al. (2024b) investigated the application of RGB cameras, 3D laser scanning, and stereo vision systems for collecting point cloud data and extracting anatomical features, including length, height, girth, and area. These features can serve as inputs for AI-based growth models, replacing manual measurements with automated, repeatable assessments conducted without animal handling. Such systems are increasingly explored for use in both confined housing and open-grazing systems.

In poultry production, imaging techniques have been applied for the automated acquisition of body dimensions and weight prediction (Benicio et al., 2021). More recently, AI models enable faster acquisition of these variables for near real-time assessment. These tools provide a non-invasive alternative to manual weighing and can support more frequent assessments of flock development. Lyu et al. (2023), for example, evaluated the use of ML algorithms to predict broiler body weight based on image-derived measurements collected on-farm. Their study demonstrated that these models could achieve high predictive accuracy under experimental conditions, indicating potential for further development into practical monitoring tools.

Beyond body weight and size, AI tools are being integrated into milk yield and productivity monitoring platforms. These systems combine data from robotic milkers, activity monitors, environmental sensors, and feeding systems. AI models are used to detect anomalies in milk production related to health disorders (e.g., mastitis), environmental stress (e.g., heat), or nutritional imbalances. Tian et al. (2024) and Ozella et al. (2023) noted that combining multiple sensor inputs with ML algorithms improves the timeliness and accuracy of detecting production-related deviations compared to traditional threshold-based alerts.

In egg production systems, DL models are being explored for automated egg counting, quality grading, and defect detection. These technologies aim to streamline post-laying processing and enhance product quality consistency. For instance, Yang et al. (2023) developed a computer vision-based system that achieved up to 94.8% accuracy in classifying eggs into categories such as intact, cracked, bloody, floor, and non-standard, while also predicting egg weight using a combination of convolutional neural networks and random forest algorithms. Similarly, Huang et al. (2023) proposed a video-based detection model that utilizes an improved YOLOv5 algorithm combined with ByteTrack for the real-time detection of broken unwashed eggs in dynamic scenes, achieving a detection accuracy of 96.4%. Further validation and integration into commercial operations remain necessary to realize these benefits.

At the broader farm level, AI technologies are increasingly being incorporated into decision support systems (DSS) that integrate health, feeding, reproduction, productivity, and environmental data to support real-time and predictive decision-making. These systems aim to streamline complex data flows into actionable insights. They use performance dashboards, alerts, and forecasting models. Distante et al. (2025) emphasized the central role of AI in enabling automated and adaptive DSS architectures, particularly through integrating machine learning pipelines with sensor networks. Niloofar et al. (2021) further noted that data-driven DSS can improve animal health and welfare while supporting greenhouse gas mitigation strategies. Their reviews emphasize the importance of interoperable data architectures and the growing interest in multimodal, AI-powered decision frameworks to achieve productivity and sustainability goals in livestock systems.

AI-based anomaly detection is an emerging application in livestock production monitoring. These systems typically utilize ML algorithms trained on historical time-series data such as milk yield, growth trajectories, or feed intake to identify deviations from expected patterns. Rather than replacing existing thresholds, these models aim to provide earlier or more context-sensitive alerts that may indicate underlying issues such as illness, suboptimal nutrition, or environmental stressors. For instance, Guien et al. (2025) developed an anomaly detection algorithm using wavelet transform features to identify deviations in cow activity, enabling early detection of disease or estrus. Similarly, Michielon et al. (2024) presented an AI-enhanced monitoring framework that integrates DL models to assess animal welfare metrics, facilitating timely interventions. Most anomaly detection models remain in research or pilot stages and require validation under diverse commercial conditions before broad adoption.

## Key Challenges and Limitations

Despite rapid advances in technology, the widespread integration of AI in livestock production remains limited. While numerous academic studies and pilot projects have demonstrated the potential of AI systems to enhance monitoring, decision-making, and efficiency, real-world implementation across diverse farming systems continues to evolve. Key barriers include technical constraints, limited infrastructure, data and privacy concerns, cultural and operational challenges, and the need for user-centered design. Addressing these interconnected issues will be essential to ensure that AI tools are inclusive, practical, and truly supportive of long-term sustainability in animal agriculture. Table 2 summarizes the major challenges for AI adoption in animal farming systems, the affected stakeholders, and proposed mitigation strategies.

**Table 2.** Major challenges and potential research directions for AI adoption in animal farming systems

Challenge area	Specific challenge	Description	Affected stakeholders	Example solution or response
**Data and Model Challenges**	Data quantity and quality	Lack of large, diverse, labeled datasets; sensor noise; limited event variability	Researchers, developers	Development of open-access annotated datasets (e.g., PigLife, MultiCamCows2024)
	Rare event representation	Imbalance in datasets for detecting health/reproduction cues like disease onset	AI developers	Synthetic data generation, sampling methods
	Model transferability	Domain shift limits generalization across breeds, housing, and environments	Researchers, integrators	Transfer learning, domain adaptation, federated learning
	Lack of general benchmarks	Few standardized tasks or datasets for livestock AI evaluation	Research community	Community challenges, benchmarking platforms
	Explainability and trust	DL models function as black boxes, hindering trust in alerts and decisions	Farmers, vets, regulators	Explainable AI (XAI) techniques like SHAP and LIME
	Limited user interfaces for interpretation	Users cannot easily view what AI systems are “seeing” or how they reason	Producers, advisors	Visual analytics, dashboards with transparent justifications
**Technical & Infrastructure Constraints**	Sensor reliability	Damage or failure due to dust, moisture, or animal contact	Farmers	Rugged hardware, automated diagnostics
	**Sensor detachment or calibration issues**	Wearables dislodge or drift, creating gaps or false data	Farmers	Design improvements, embedded calibration alerts
	**Connectivity limitations**	Many rural areas lack broadband to support cloud-based AI	Small farms, rural users	Edge AI, offline-capable tools, LoRa/mesh networks
	Edge computing hardware costs	Real-time edge devices are still expensive or limited in processing power	Farmers, integrators	Lightweight architectures (e.g., TinyML, model pruning)
	System integration & interoperability	Incompatible software/hardware from different vendors	Tech providers, integrators	Open-source APIs, industry data standards
**Ethical, Legal, and Social Concerns**	Data ownership and governance	Unclear ownership of sensor-collected data; risk of misuse	Farmers	Transparent governance, data sharing agreements, Ag Data Transparent principles
	Cybersecurity risks	Farm data may be vulnerable to breaches or misuse	Producers, tech providers	Encrypted storage, farm-specific access controls
	Bias and fairness	AI tools may be trained on high-performing farms, not generalizable	Underserved farm types, smallholders	Diverse training data, cross-site validation
	Reduced human-animal interaction	Over-automation risks loss of daily contact important for welfare monitoring	Caregivers, animals	Hybrid systems that prompt visual inspection, staff alerts
	Welfare trade-offs in optimization	Algorithms may prioritize throughput over animal comfort if not constrained	Producers, policy advocates	Embed welfare thresholds in optimization routines
	Lack of regulation or standards	No legally enforceable ethics or performance standards for livestock AI	Government, industry	Industry consortia, regulatory frameworks, third-party audits
**Adoption and Training Gaps**	Technological unfamiliarity	Many producers lack background in AI/data systems	Farmers	Extension programs, visual training tools
	Perceived complexity of tools	Black-box nature, unfriendly interfaces discourage use	End-users	User-centered design, mobile interfaces
	Fear of job displacement	Concern that AI may replace labor-intensive roles	Farmworkers	Reframing AI as augmentative, retraining initiatives
	Cost and risk aversion	Capital costs and uncertainty about return delay adoption	All producers	Demonstration farms, phased investment plans
	Lack of participatory design	Tools built without farmer input fail to meet real-world needs	Farmers, developers	Co-design workshops, iterative prototyping

### Data and model challenges

#### Data quantity and quality

The effectiveness of AI systems in livestock production critically depends on the availability of large, diverse, and well-annotated datasets, especially for supervised learning approaches. Yet, data limitations remain one of the most persistent barriers in this field. High-resolution, labeled datasets are scarce, and they are often fragmented across farms and institutions and rarely standardized for sensor types, annotation protocols, or sampling frequency. Sensor data is frequently affected by environmental noise, inconsistent calibration, and animal movement. These issues further complicate model training and validation (Stygar et al., 2021; Tedeschi et al., 2021).

Additionally, datasets often lack representation of rare but biologically significant events such as illness onset, aggressive interactions, or reproductive anomalies. These imbalances reduce model reliability and can lead to poor generalization during real-world deployment. In particular, behavior-based datasets are typically unstructured and contain few clearly labeled edge cases, which makes it challenging to extract reliable behavioral patterns (McVey et al., 2023).

To address these issues, several research groups have developed open, annotated datasets to support AI development in livestock contexts. For instance, the PigLife dataset offers video clips and images across various pig production phases, including breeding, gestation, farrowing, weaning, nursery, growth, and finishing stages, with annotations for object identification, pig posture, and behavior labels (Li et al., 2024b). Similarly, MultiCamCows2024 provides a multi-view image dataset comprising over 100,000 images of Holstein-Friesian cattle captured with ceiling-mounted cameras over seven days on a working dairy farm, which facilitates biometric identification and behavior analysis (Yu et al., 2024). These initiatives are essential for benchmarking AI tools, fostering algorithm development, and promoting reproducibility across research groups, and they will require broader investment and collaboration to expand across species, management systems, and production conditions.

#### Model transferability and generalization

A significant challenge in deploying AI systems across diverse livestock farming environments is the limited transferability of models. Models trained in data from specific breeds, housing types, or sensor systems often perform poorly when applied to different contexts. For instance, a lameness detection model developed for Holstein cows in free-stall housing may not generalize to Jersey cows in pasture-based setups because locomotion patterns, backgrounds, and data quality differ. This issue, known as domain shift, complicates scalability and reduces the reliability of AI systems outside their original training domain.

To address this, researchers are exploring transfer learning, domain adaptation, and federated learning, which aim to improve model robustness across different production environments. For example, unsupervised domain adaptation methods have been employed to mitigate sensor variability and interspecies heterogeneity in animal activity recognition tasks (Ahn et al., 2023). Additionally, federated learning frameworks, such as FedAAR, have been developed to enable collaborative model training across farms without sharing sensitive data, which preserves privacy and enhances model generalization (Mao et al., 2022). However, these techniques are still largely experimental in livestock contexts, and practical implementation remains limited because of high technical complexity, computational demands, and the need for ongoing updates as farm conditions evolve.

#### Explainability and trust

The complexity and “black box” nature of many AI algorithms, particularly DL models, present significant barriers to adoption in livestock management (Tedeschi, 2019). Stakeholders, including farmers, veterinarians, and regulators, require clear insights into how AI systems generate specific predictions or recommendations, particularly in critical areas such as animal health, reproduction, and welfare. A lack of transparency can lead to skepticism and reluctance to rely on these tools.

The field of explainable artificial intelligence (XAI) has emerged to address these concerns, and develops methods that make AI decision-making processes more transparent and interpretable (Hoxhallari et al., 2022). Techniques such as SHapley Additive exPlanations (SHAP) and Local Interpretable Model-Agnostic Explanations (LIME) are increasingly used to elucidate the contributions of input features to model outputs, thereby enhancing user understanding and trust (Cartolano et al., 2024).

In the context of PLF, integrating XAI methods can provide stakeholders with comprehensible explanations of AI-driven decisions, which can facilitate better acceptance and more effective interventions. Useful deployment also depends on interfaces that present explanations clearly to farmers and veterinarians. For instance, applying SHAP and LIME to models predicting animal health outcomes could help veterinarians and farmers understand the underlying factors influencing predictions and support more informed decision-making.

However, practical implementation of XAI in livestock systems remains at an early stage. Challenges include model complexity, the need for user-friendly interfaces, and integration with existing farm management practices. Ongoing research and development are essential to tailor these explainability tools to the specific needs and capabilities of agricultural stakeholders.

### Technical and infrastructure constraints

#### Sensor reliability and maintenance

Sensors are fundamental components of AI-driven livestock systems; however, their reliability often suffers under the harsh and variable conditions typical of farm environments (Stygar et al., 2021; Tedeschi et al., 2021). Factors such as dust, moisture, temperature fluctuations, animal interference, and improper equipment handling can severely degrade sensor accuracy and reduce device lifespan. Devices like wearable sensors may frequently detach or become damaged due to animal behavior, creating gaps and erroneous readings that compromise the accuracy of AI models (Stygar et al., 2021; Neethirajan, 2024). Ensuring continuous, high-quality data collection requires regular sensor calibration, maintenance, and troubleshooting. Unfortunately, producers often lack the technical expertise, resources, or motivation necessary for consistent sensor management, and this exacerbates data reliability issues (Tedeschi et al., 2021; Greig et al., 2023; Neethirajan, 2024). Efforts to improve sensor durability and robustness include ruggedized hardware and automated diagnostic systems, such as the one proposed by Schulthess et al. (2024), yet many of these options remain relatively costly or underexplored in livestock contexts (Tedeschi et al., 2021).

#### Connectivity and processing limitations

Reliable internet connectivity remains a significant challenge for many livestock operations, particularly in rural areas. The Federal Communications Commission (FCC, 2021) reported that in 2019, approximately 17% of people living in rural areas in the United States lacked broadband access, compared to 1% in urban areas. This lack of connectivity limits the implementation of cloud-based AI systems that require stable internet connections for data processing and storage. Uploading high-resolution video or audio data for real-time AI processing is often impractical in regions with limited bandwidth, which limits data use and slows system response.

Edge computing, which processes data locally on the farm rather than sending it to centralized servers, offers a promising solution to these connectivity challenges. Edge computing enables real-time data analysis and reduces dependence on internet connectivity, which enhances the efficiency of AI-driven livestock management systems. However, deploying and maintaining edge computing infrastructure requires sophisticated hardware and stable power sources, and these requirements can pose significant technical and financial burdens for producers. Moreover, integrating locally processed data with central databases for benchmarking and long-term analytics remains a complex and challenging task.

#### Integration and interoperability

Livestock operations often use a mix of technologies from multiple vendors, including automated milking systems, RFID readers, climate control units, and feeding equipment. Many of these systems lack standardized communication protocols, which creates interoperability issues that complicate data integration and hinder the development of comprehensive AI-driven decision support systems. The absence of standardized data formats and communication protocols also blocks progress toward unified AI platforms and dashboards. Recent efforts, such as those described in CAST (2025), recommend adopting frameworks such as the FAIR (Findable, Accessible, Interoperable, and Reusable) data principles and AgGateway’s ADAPT (Agricultural Data Application Programming Toolkit, 2019) to enhance interoperability and enable efficient data exchange across farm systems. Additionally, work on open-source platforms, standardized APIs, and interoperability frameworks is ongoing, yet efforts remain fragmented and thinly supported. Enhanced industry-wide cooperation and regulatory support are essential for progress in this domain (Bahlo et al., 2019; Habib et al., 2025).

### Ethical, legal, and social considerations

#### Data privacy, cybersecurity, and ownership

The proliferation of AI technologies in livestock farming has produced highly granular data, raising significant concerns about data privacy, ownership, and security. Farmers often face uncertainty about who holds rights to the data collected from commercial sensors, how this data can be shared or sold, and the implications for regulatory oversight or competitive advantage. Many producers fear potential data misuse by insurers, competitors, or regulators, particularly if the data reveal operational shortcomings or animal welfare issues and harm their reputation (Kaur et al., 2022).

The lack of clear legal frameworks defining data ownership in agriculture exacerbates these concerns. Agricultural technology providers (**ATPs**) often have extensive control over farm data under complex service agreements, sometimes without farmers fully understanding the implications. As a result, farmers may inadvertently relinquish control of their data, which limits their ability to manage its use and distribution (Wiseman et al., 2019; Kaur et al., 2022).

There is a pressing need for transparent data governance frameworks that clearly define ownership rights, usage permissions, and data anonymization practices to address these challenges. Such frameworks would help build trust among stakeholders and facilitate the broader adoption of AI technologies in agriculture. Initiatives like the American Farm Bureau Federation’s “Privacy and Security Principles for Farm Data” (established in 2014 and updated in 2024 by the Ag Data Transparent organization; Ag Data Transparent, 2024) aim to establish guidelines for responsible data management, emphasizing the importance of farmer control over their data. However, these principles are voluntary and lack the enforceability of formal legislation.

Collaborative efforts among farmers, technology providers, policymakers, and researchers are crucial for developing and implementing robust data governance policies that protect farmers’ interests and promote innovation in AI-driven livestock management.

In addition to concerns about ownership and privacy, integrating AI and autonomous systems into agriculture introduces critical cybersecurity risks. The potential for cyberattacks to disrupt AI-enabled agricultural systems, including autonomous equipment and decision-support tools, is real (CAST, 2025). Threat actors could exploit software or communications infrastructure vulnerabilities, compromising operations, data integrity, and food security. Effective mitigation requires comprehensive cybersecurity strategies to safeguard the benefits of digital agriculture.

#### Bias, fairness, and animal ethics

While AI technologies offer significant potential for improving livestock management, they also raise important ethical and fairness considerations that require proactive attention. One concern is that models trained on data from high-performing or well-resourced farms may not generalize well to other production systems. This could inadvertently introduce or reinforce biases, leading to unequal performance across farm types and widening existing technological divides (Albergante et al., 2025). Such disparities exemplify the need for diverse, representative datasets and cross-environment validation protocols.

Additionally, increasing automation of routine tasks may reduce the frequency and quality of human-animal interactions, which play a recognized role in supporting animal welfare. Studies have shown that regular positive contact improves animal behavior and productivity, while its absence may hinder early detection of welfare issues or reduce empathetic caregiving (Cornou, 2009; Zulkifli, 2013). Although automation can alleviate labor burdens, systems must be designed to support, rather than replace, routine visual checks and care practices by trained staff.

There are also broader concerns that AI tools could contribute to intensified production systems that prioritize output over well-being when implemented without clear animal welfare guidelines. For example, optimization algorithms designed to increase throughput could unintentionally lead to conditions like overcrowding or neglect of individual health needs (Bossert and Coeckelbergh, 2024). However, these risks can be mitigated through animal-centric design principles into AI development. Strategies such as embedding welfare thresholds into optimization models, using sensor systems for real-time individual health monitoring, and requiring human oversight in decision loops have been proposed to balance productivity with welfare goals (Webber et al., 2022; Neethirajan, 2024; Rosati, 2024). Mitigation also benefits from engagement with ethicists, animal welfare experts, producers, and policymakers to ensure responsible and equitable deployment across the livestock industry.

#### Adoption resistance and training gaps

The integration of AI technologies into livestock systems brings significant human-centered challenges. Many producers exhibit skepticism or hesitation toward adopting AI tools due to unfamiliarity, concerns about the effectiveness of technology, and fears of job displacement or erosion of traditional farming knowledge. Economic constraints, inadequate infrastructure, and limited technological knowledge further exacerbate these barriers, particularly among small- and medium-sized farms (Dibbern et al., 2024).

Bridging these gaps requires comprehensive capacity-building strategies. Targeted educational programs and extension services can build technological literacy and show the practical benefits of AI applications in livestock management (Atapattu et al., 2024). Participatory design initiatives that involve farmers in the development and implementation of AI tools help tailor technologies to the specific needs and contexts of end users (Mallinger et al., 2024). Demonstration farms showcasing AI technologies can serve as tangible examples of successful integration, fostering trust and encouraging wider adoption.

User-centered design is particularly essential. Ensuring that AI tools are intuitive, adaptable, and compatible with existing farm management practices could lower the learning curve and increase user engagement (Ajibola et al., 2024). Such strategies would support the adoption of AI technologies and promote sustainable and efficient livestock farming practices.

Finally, a growing concern is that increased automation and reliance on AI could erode traditional farming skills and reduce the transfer of experiential knowledge across generations (CAST, 2025). Maintaining a balance between technological assistance and foundational knowledge is essential for long-term resilience, adaptability, and independence within the agricultural workforce (Tedeschi, 2019).

## Emerging Trends and Research Opportunities

As AI technologies mature and the livestock industry continues to digitize, several emerging trends are likely to significantly influence future research and development. These potential innovations reflect a shift from isolated tools to integrated, context-aware, and adaptive systems. At the same time, researchers are increasingly exploring multisensory integration, novel computational techniques, and participatory approaches that involve producers directly in system design and deployment. This section discusses some key trends and research opportunities that might shape AI advancements in livestock farming.

### Multimodal sensor fusion and digital twins

The increasing availability of diverse sensors in livestock farming presents opportunities for multimodal sensor fusion, which enhances the accuracy of AI predictions, reduces false alarms, and captures complex physiological states and behaviors. For instance, integrating accelerometry and Global Navigation Satellite System (GNSS) data has been shown to improve the classification of animal behaviors, such as walking and drinking, when movement patterns are combined with location information (Arablouei et al., 2023). Similarly, fusing acoustic and linguistic data has demonstrated effectiveness in decoding dairy cow vocalizations, providing insights into their emotional states and welfare (Jobarteh et al., 2024).

A promising research avenue involves the development of digital twin technologies, which are virtual representations of animals or entire farm systems continuously updated with real-time sensor data. Building on decades of simulated-population work in animal science to study various aspects of the production system, such as nutrient flows, herd dynamics, and disease spread (Gouttenoire et al., 2011; Black, 2014), the new contribution of digital twins is their ability to integrate multimodal sensor data and update system states in real-time. These AI-enabled, data-driven twins can simulate various management scenarios, including health risks, productivity outcomes, and environmental impacts, thereby aiding decision-making processes (Neethirajan and Kemp, 2021). Implementing digital twins in livestock farming has the potential to improve animal health and welfare, optimize feed rations, and reduce operational costs when inefficiencies are identified (Symeonaki et al., 2024). However, achieving effective digital twins requires advancements in data integration frameworks, real-time processing capabilities, and robust biological modeling to ensure the creation of meaningful simulations.

### Edge AI and real-time inference

As AI applications in livestock farming evolve, Edge AI is emerging as a promising direction for enabling real-time decision-making on the farm. Unlike traditional cloud-based approaches, Edge AI involves deploying models directly onto local hardware devices, so data is processed at the source. This can address challenges such as unreliable internet connectivity, latency, and data privacy concerns, which are common barriers in rural production settings.

Prototype systems have demonstrated how Edge AI could support on-farm monitoring of animal health and behavior. For example, local devices that analyze data from wearable sensors or environmental cameras may be used to flag behavioral anomalies, such as signs of aggression or discomfort. While these applications are still in the early stages of development, they suggest the potential for more responsive and automated interventions to improve animal welfare and management efficiency (Arablouei et al., 2023).

To make Edge AI viable in livestock contexts, researchers are exploring lightweight model architectures such as MobileNet and Tiny YOLO, designed to run on low-power devices. Additional strategies, like model pruning, quantization, and other compression techniques, can reduce computational demands without significantly compromising performance. These developments make it more feasible to run AI models on affordable, ruggedized hardware suited to agricultural environments (Avanija et al., 2024).

Federated learning complements this direction by training models locally and sharing only model updates, not raw data, with a central server. This approach preserves data privacy while enabling collective model improvement across geographically distributed operations. In combination with edge inference, reported benefits include greater resilience, stronger privacy protections, and more context-relevant performance for real-time monitoring and decision support (Dembani et al., 2025). Current livestock research combines Edge AI and federated learning in experimental systems, and deployment at production scale remains limited.

### Integration with genomics, nutrition, and climate data

Emerging research suggests that integrating genomic, nutritional, and environmental data into AI models holds considerable promise for enhancing predictive capabilities and informing management strategies in livestock farming. Although current AI applications often operate within a single domain, such as health monitoring or behavioral analysis, broader integration is feasible through multimodal data fusion strategies that combine biological measurements with production and environmental records to produce context-aware predictions.

This approach integrates information from multiple sources, including genotypes, sensor-based phenotypes, feed intake proxies, and climate conditions, within a unified analytical framework. Fully integrated systems that robustly attribute performance changes to genetics, heat stress, or nutrition are still rare, but context-aware models are feasible and have shown promise for improved biological interpretation when genomics are modeled jointly with recorded environmental exposures such as temperature–humidity index (THI) and nutrition-related indicators such as mid-infrared–derived energy balance and dry matter intake estimates. Momentum toward integrated systems is accelerating in precision livestock research, supported by advances in multimodal learning, interoperable farm data platforms, such as Dairy Brain, and standardized trait ontologies, such as the Animal Trait Ontology for Livestock (ATOL) (Golik et al., 2012; McParland et al., 2014; Garner et al., 2016; Cabrera and Fadul-Pacheco, 2021; Aguilar-Lazcano et al., 2023; Kaur et al., 2023; Landi et al., 2023; McWhorter et al., 2023; Brito et al., 2025; McFadden et al., 2025).

Environmental and climatic variables are increasingly incorporated into AI systems due to their strong influence on animal performance, health, and welfare. In this context, “environment” refers to local and immediate conditions within the production system, such as temperature, humidity, ventilation, air quality, stocking density, and housing design. In contrast, “climate” describes broader and longer-term weather patterns, including seasonal heat trends and extreme weather variability, which shape long-term risk exposure for livestock systems. AI tools are being developed to integrate these environmental and climatic inputs with animal-level sensor data, thereby improving the early detection of stress responses, such as heat stress or disease risk, and supporting adaptive management strategies under variable climatic conditions (Reeves et al., 2015; Derner and Augustine, 2016; Chapman et al., 2023; Rebez et al., 2024; Woodward et al., 2024; Eckhardt et al., 2025).

Incorporating genomic data into AI models is being explored as a way to improve predictions related to disease susceptibility and support precision breeding strategies. Machine learning algorithms have been applied to capture nonlinear effects and complex interactions within genomic datasets. However, results remain mixed as several comparative studies have reported that deep learning methods do not consistently outperform linear additive models for genomic prediction, especially when training datasets are limited. Even with these limitations, continued research is evaluating whether integrating genomic data with phenotypic and environmental information through multimodal AI frameworks may yield more robust prediction accuracy for features of the genome (Abdollahi-Arpanahi et al., 2020; Montesinos-López et al., 2021; Chafai et al., 2023; Lourenço et al., 2024; Džermeikaitė et al., 2025; Klingström et al., 2025).

Similarly, AI-driven nutritional models that integrate dietary inputs, nutrient digestibility, and microbiome profiles are under development to better inform individualized feeding strategies. These systems may contribute to optimizing feed efficiency and improving animal health; however, most remain at a research or prototype stage, rather than undergoing widespread adoption (Tedeschi, 2022; Pomar & Remus, 2023).

Realizing the full value of this kind of integrated approach requires overcoming key challenges in multi-scale data harmonization, causal inference modeling, and the development of robust datasets that link genetic, phenotypic, and environmental data. Continued interdisciplinary research and stakeholder collaboration will be critical to making these complex systems practical and impactful for producers.

### Human-centered design and participatory AI

Human-centered design (HCD), participatory design, and group model building (GMB) are increasingly recognized as essential for improving the usability and adoption of AI technologies in livestock systems. Historically, limited stakeholder involvement during AI system development led to a poor fit with operational realities and lowered adoption (McGrath et al., 2024).

Human-centered design emphasizes iterative design centered on the needs, constraints, and workflows of end-users. In livestock contexts, this ensures AI tools are intuitive, robust, and aligned with the daily practices of producers and veterinarians (Garard et al., 2024). Participatory design extends this approach through direct stakeholder involvement in co-design activities such as workshops and prototyping. It has been used in agriculture to enhance alignment between technology and farm-level decision-making, including integration with constraint programming to reflect farmer priorities (Challand et al., 2025).

Building on participatory design, GMB introduces a structured, system dynamics-based approach to collaborative modeling. GMB uses facilitated sessions to help stakeholder groups define problems, develop shared mental models, and simulate policy or management scenarios (Vennix, 1996; Andersen et al., 2007). Unlike traditional modeling that separates analysts from end-users, GMB treats model construction as a participatory process, strengthening system understanding and shared ownership of outcomes. As described in Andersen et al. (2007), models built through GMB serve a dual role. They function as formal simulations of policy systems and as boundary objects that support dialogue, surface assumptions, and structure decision-making. A central benefit of GMB is that it creates space for experiential knowledge from producers, which is often overlooked in datasets but is essential for understanding real production constraints and management logic. Incorporating producer knowledge into model structure fosters trust and increases the likelihood that resulting AI tools will be viewed as credible and relevant to on-farm decision-making. This dual identity enhances analytical rigor and stakeholder engagement, making GMB especially well-suited for guiding the integration of AI in complex, context-rich environments.

Group model building has long been used in agricultural development to improve collective understanding of complex issues like animal health, resource use, and farm economics (Gouttenoire et al., 2013). Recently, this approach has expanded to incorporate AI models into the collaborative process, not just as analysis tools but as active “co-modelers.” In these emerging hybrid formats, AI tools simulate real-time projections and surface patterns in complex datasets or evaluate scenario trade-offs while users supply contextual knowledge and refine inputs.

Framing AI tools as collaborative modeling partners, rather than black-box decision engines, improves interpretability and trust. Trust is strengthened when producers can see their input reflected in model assumptions and outputs, reinforcing transparency and model legitimacy. When users help define model logic and interrogate outputs, they are more likely to integrate the tool into routine decision-making. Explainable AI techniques, such as SHAP and LIME, support this approach as they clarify how input data influences recommendations (Hoxhallari et al., 2022; Mallinger et al., 2024).

Finally, expanding educational resources (e.g., simulation-based training, intuitive mobile interfaces, peer-led workshops) and encouraging shared learning across design traditions, as GMB practice has done in Europe and the U.S., are critical to mainstreaming participatory AI in agriculture to ensure AI technologies are accessible and actionable for producers at various levels of digital literacy (Prajapati et al., 2025). The continued refinement of collaborative modeling methods and evaluation of their effectiveness are essential for scaling inclusive AI innovation in livestock systems (Andersen et al., 2007).

## Conclusion and Future Perspectives

Artificial intelligence has the potential to significantly reshape livestock farming in ways that were scarcely imagined a decade ago. From enhancing early disease detection and estrus monitoring to optimizing feeding strategies and behavior tracking, AI-driven systems are beginning to redefine animal care and management. These technologies hold promise not only for improving productivity and animal welfare but also for addressing labor shortages, reducing environmental impacts, and strengthening the resilience of livestock systems in response to global challenges such as climate change and food insecurity.

Nevertheless, significant barriers hinder widespread adoption. These challenges include data availability and quality, model generalization across diverse farming contexts, technical infrastructure constraints, ethical concerns, and socio-cultural acceptance. Limited training datasets, fragmented and proprietary technologies, sensor reliability issues, and insufficient rural connectivity present substantial hurdles. Additionally, issues surrounding algorithmic transparency, explainability, data governance, and user trust have a critical impact on stakeholder acceptance.

Addressing these challenges requires more than technical innovation alone; it requires holistic, systems-oriented strategies that are sensitive to the biological, social, and economic complexities of animal agriculture. Realistically, adoption depends on strengthening the broader PLF ecosystem, which involves linking producers, vendors, connectivity, open standards, and service capacity, so that point solutions operate as a coherent whole. Future AI developments could benefit from creating adaptable, interpretable models tailored to diverse farm contexts and from investments in infrastructure improvements such as robust rural connectivity and effective edge computing solutions. Developing clear guidelines for data ownership, privacy, and transparency will also be essential in building trust among producers and stakeholders.

Promising research directions highlighted in this review, such as multimodal sensor fusion, digital twin technologies, edge computing, genomics and climate data integration, and human-centered design approaches, include avenues to foster more comprehensive and responsive livestock management systems. These advancements depend on overcoming current technical and methodological challenges, such as data integration, real-time processing, lightweight modeling architectures, and effective stakeholder engagement.

Interdisciplinary collaboration could significantly enhance the success of AI in livestock farming. Policymaking supportive of open data initiatives, interdisciplinary funding opportunities, and extensive capacity-building programs for end-users could further strengthen the adoption ecosystem.

As AI becomes more embedded within livestock systems, the human role is likely to evolve rather than diminish, transforming producers into informed system managers and insightful data interpreters. AI technologies, therefore, might serve as partners, amplifying human expertise rather than replacing it, thus fostering more sustainable, ethical, and productive animal agriculture practices.

Although numerous challenges remain, the opportunities are substantial. Real progress now depends on moving from pilots to validated, farm-ready systems, prioritizing interpretability and trust, and building open, interoperable data ecosystems with clear governance. In practical terms, the field should publish reproducible, context-aware baselines, invest in standards that allow sensors and software to interoperate, co-design tools with producers using group model building, and judge success by farm-relevant outcomes such as timely alerts, avoided treatments, and reduced repeat breedings. Taken together, these steps can make AI not only possible but reliably useful in daily production.

## Abbreviations:

ADAPT: Agricultural Data Application Programming Toolkit
AI: artificial intelligence
ANN: artificial neural network
AR: augmented reality
ATOL: Animal Trait Ontology for Livestock
CAST: Council for Agricultural Science and Technology
CNN: convolutional neural network
CV: computer vision
DL: deep learning
DSS: decision support system(s)
FAIR: Findable, Accessible, Interoperable, and Reusable
FCC: Federal Communications Commission
GMB: group model building
GNSS: Global Navigation Satellite System
GPS: Global Positioning System
HCD: human-centered design
HGS: Horse Grimace Scale
HIMM: hybrid intelligent mechanistic model
IoT: Internet of Things
LIME: Local Interpretable Model-agnostic Explanations
LoRaWAN: Long Range Wide Area Network
LPS: local positioning system
LSTM: long short-term memory
ML: machine learning
MPE: mean percentage error
PLF: precision livestock farming;
R-CNN: region-based convolutional neural network;
RFID: radio-frequency identification
RGB: red, green, blue;
RGB-D: red, green, blue + depth;
RNN: recurrent neural network
ROI: region of interest
SHAP: SHapley Additive exPlanations
SNA: social network analysis
THI: temperature–humidity index
XAI: explainable AI
YOLO: You Only Look Once
5G: fifth-generation mobile network
